# The Immunological Purity Theory: a testable framework linking peri-menstrual endometrial immune transition to receptivity and implantation, with exploratory implications for neurodevelopment

**DOI:** 10.3389/fimmu.2026.1957528

**Published:** 2026-10-13

**Authors:** Budor H. Saigh

**Affiliations:** Department of Special Education, College of Education, Umm Al-Qura University, Makkah, Saudi Arabia

**Keywords:** autism spectrum disorder, developmental programming, endometrial immune variation, epigenetic reprogramming, hypothesis and theory, implantation, maternal immune activation, neurodevelopment

## Abstract

The transition from menstruation to endometrial regeneration is a dynamic biological interval involving inflammatory resolution, immune-cell regulation, epithelial repair, vascular remodeling, and endocrine-dependent tissue renewal. Although these processes are individually well established, it remains unclear whether measurable variation in the peri-menstrual endometrial immune transition predicts subsequent endometrial differentiation, receptivity, or implantation within the same cycle. This unresolved question defines the primary focus of the present framework. This article proposes the Immunological Purity Theory, a testable conceptual framework in which *immunological purity* refers to the transition from menstrual-associated inflammatory and reparative activity toward a regulated endometrial state compatible with subsequent receptivity. The term does not denote sterility, absence of immune activity, complete absence of inflammation, or an established clinical condition. The core hypothesis proposes that interindividual and intraindividual variation in the timing, coordination, or characteristics of this immune–repair transition may prospectively relate to the subsequent trajectory of endometrial regeneration, hormone-dependent differentiation, receptivity, and implantation. The framework does not assume that menstrual inflammation persists until implantation. Rather, it proposes a testable within-cycle relationship between an early endometrial immune–repair phenotype and the later receptive state of the same tissue. This distinction is important because menstruation occurs during the early follicular phase, whereas implantation occurs during the mid-secretory phase. Accordingly, prospective studies are required to determine whether early-cycle immune–repair characteristics provide reproducible information about later endometrial function beyond normal cycle-dependent variation. Placental adaptation, epigenetic programming, and neurodevelopment are presented as sequential downstream extensions of the core reproductive hypothesis rather than established consequences of peri-menstrual immune variation. Current evidence provides biological plausibility for individual components of these pathways but does not validate the complete proposed sequence, and no direct evidence currently links peri-menstrual endometrial immune variation to autism spectrum disorder. The framework therefore prioritizes characterization of reproducible peri-menstrual immune–repair phenotypes and their prospective relationships with receptivity and implantation before investigation of more distal developmental outcomes. The Immunological Purity Theory is presented as a falsifiable research framework requiring direct empirical validation and should not be interpreted as clinical guidance regarding conception timing or neurodevelopmental risk.

## Graphical Abstract

**Figure:**
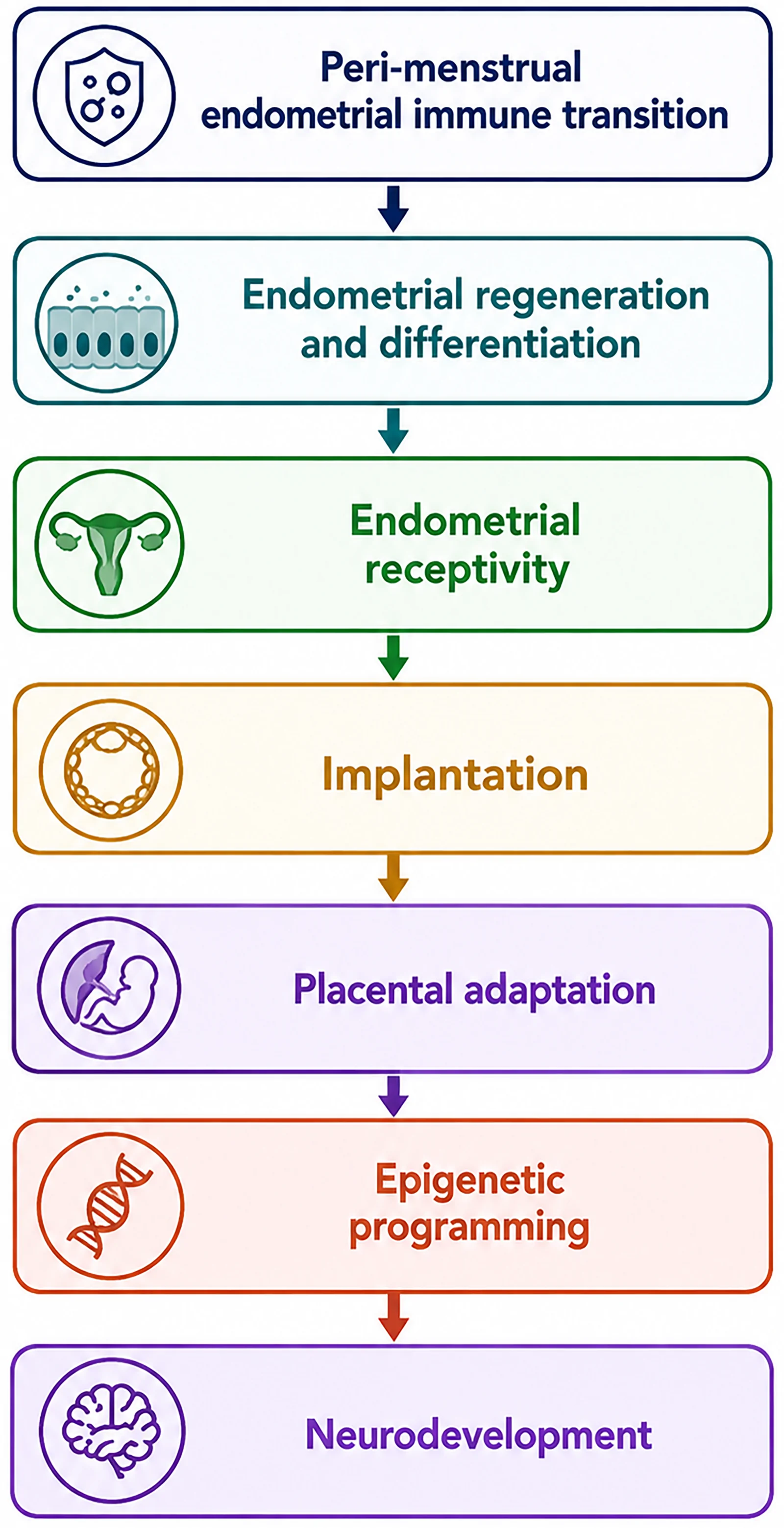
Simplified overview of the Immunological Purity Theory (IPT). The proposed core reproductive sequence progresses from the peri-menstrual endometrial immune transition through endometrial regeneration and differentiation to receptivity and implantation within the same menstrual cycle. Placental adaptation, epigenetic programming, and neurodevelopment represent progressively downstream exploratory extensions rather than established consequences of the core hypothesis. All IPT-specific relationships remain hypothetical and require independent empirical validation.

## 1 Introduction

Human implantation occurs within a dynamic endometrial environment shaped by endocrine signaling, tissue remodeling, vascular adaptation, immune-cell activity, and embryo–maternal communication. These processes are temporally coordinated across the menstrual cycle and cannot be understood as isolated events. Menstruation itself is a physiological inflammatory and reparative process, whereas successful implantation later in the cycle requires a regulated immune environment capable of supporting endometrial differentiation, embryo attachment, trophoblast invasion, and maternal–fetal immune adaptation (1–4).

Progesterone withdrawal at the end of the luteal phase initiates endometrial breakdown through coordinated vascular, inflammatory, and extracellular-matrix changes. Leukocyte recruitment, cytokine and chemokine signaling, matrix metalloproteinase activity, hypoxia-associated responses, and shedding of the functional endometrial layer occur alongside rapidly initiated repair mechanisms (2, 4). Re-epithelialisation, vascular repair, extracellular-matrix remodeling, and tissue regeneration begin during menstruation and normally restore endometrial integrity without scarring (5, 6).

This rapid repair is important to the present hypothesis. The framework does not assume that visible cessation of menstrual bleeding generally precedes incomplete endometrial healing, nor does it propose that menstrual inflammation persists unchanged until implantation. Rather, it distinguishes an externally observable event—cessation of bleeding—from the underlying molecular and cellular processes involved in inflammatory resolution, tissue repair, immune-cell regulation, and subsequent endometrial regeneration. Whether women who appear clinically similar at the end of menstruation exhibit equivalent biological trajectories during this transition remains incompletely characterized.

The term peri-menstrual endometrial immune transition is therefore used in this article as an operational construct describing the coordinated shift from menstrual-associated inflammatory and reparative activity toward subsequent endometrial regeneration and immune regulation. It is not intended to define a pathological state or a binary distinction between “recovered” and “unrecovered” endometrium. Normal menstrual physiology is inherently variable, and variation in cytokine signaling, immune-cell composition, tissue repair, vascular remodeling, and endocrine responsiveness may occur both between individuals and between cycles within the same individual. The primary scientific question is whether any component of this variation is reproducible and functionally informative for later endometrial biology.

A central temporal issue must be explicitly recognized. Menstruation occurs during the early follicular phase, whereas implantation normally occurs only after ovulation, during the receptive mid-secretory phase. The proposed framework therefore does not treat menstruation and implantation as immediately adjacent biological events. Between them, the endometrium undergoes epithelial and stromal regeneration, estrogen-dependent proliferation, post-ovulatory progesterone-dependent differentiation, decidualization-related changes, and establishment of the receptive phenotype (7–10).

Accordingly, the proposed link is one of prospective biological continuity rather than persistence of menstrual inflammation. The core question is whether measurable characteristics of the early-cycle immune–repair transition reflect or predict the subsequent trajectory of regeneration and differentiation of the same endometrial tissue. Preliminary human research supports the feasibility of studying such an early-to-later cycle relationship. Immune mediators measured in menstrual material have been investigated as indicators of endometrial biology, and menstrual-blood-derived endometrial cells can retain biologically informative properties relevant to hormone responsiveness and endometrial differentiation. These observations do not demonstrate that peri-menstrual immune variation determines later receptivity; rather, they provide a rationale for prospective studies directly testing this relationship (11, 12).

Following ovulation, progesterone drives major functional changes in the estrogen-primed endometrium. Stromal differentiation, epithelial remodeling, immune-cell redistribution, vascular adaptation, and expression of adhesion molecules, cytokines, chemokines, growth factors, and other mediators contribute to establishment of the window of implantation (8, 10, 13). Controlled inflammatory signaling is integral to implantation biology; therefore, the relevant question is not whether inflammation is present or absent, but whether the timing, coordination, and resolution of immune activity are compatible with subsequent endometrial function.

The endometrial immune compartment further illustrates this complexity. Uterine natural killer cells, macrophages, dendritic cells, regulatory T cells, neutrophils, and other leukocyte populations perform context-dependent functions across the menstrual cycle and early pregnancy. Uterine natural killer cells, for example, contribute to vascular remodeling and trophoblast regulation rather than simply representing cytotoxic inflammatory cells, while macrophages participate in tissue clearance, repair, angiogenesis, and immune regulation (1, 13, 14). Consequently, the proposed peri-menstrual phenotype cannot be reduced to a single cytokine concentration or to a simple “high-versus-low inflammation” model.

Additional biological and behavioral factors may modify the peri-menstrual reproductive environment, but they should be distinguished from the core hypothesis until directly tested. One such candidate modifier is seminal exposure. Seminal fluid is biologically active and can induce local inflammatory and regulatory responses within the female reproductive tract that participate in reproductive immune adaptation (15, 16). However, whether the effects of seminal exposure differ according to the peri-menstrual endometrial immune state at the time of exposure has not been directly established. The present framework therefore treats exposure timing as a secondary, testable modifier rather than as an established cause of altered endometrial receptivity.

Microbial ecology represents another potential modifier, but interpretation requires particular caution. Associations have been reported between reproductive-tract microbial profiles, inflammatory signaling, implantation failure, and pregnancy loss (17–20). However, the endometrium is a low-biomass environment in which contamination, sampling route, sequencing methodology, and analytical thresholds can substantially influence findings. Microbial ecology is therefore considered an exploratory component of the framework rather than a necessary mechanism.

The developmental significance of the peri-conceptional environment extends beyond implantation. The Developmental Origins of Health and Disease framework and experimental maternal immune activation literature demonstrate that maternal biological conditions can influence placental function and developmental trajectories through endocrine, immune, metabolic, and potentially epigenetic mechanisms (21–24). These literatures provide important biological precedent, but they do not constitute direct evidence for the Immunological Purity Theory. In particular, evidence that maternal immune activation during established pregnancy can influence fetal development cannot be extrapolated directly to subtle peri-menstrual immune variation occurring before implantation.

This distinction is particularly important for neurodevelopment. Maternal inflammatory conditions have been associated with offspring neurodevelopmental outcomes, and experimental maternal immune activation can alter placental signaling, microglial development, neural organization, and offspring behavior (25–27). Nevertheless, causal interpretation in humans remains limited by genetic and familial confounding, exposure heterogeneity, maternal health, medication use, socioeconomic factors, and other co-exposures (28). Autism spectrum disorder is additionally highly heterogeneous and has substantial genetic contributions (29–31). No direct evidence currently demonstrates that peri-menstrual endometrial immune variation influences autism risk.

For this reason, the revised framework distinguishes a core reproductive hypothesis from its downstream exploratory extensions. The core hypothesis concerns whether measurable variation in the peri-menstrual endometrial immune transition predicts subsequent receptivity and implantation. Placental adaptation, epigenetic programming, and neurodevelopment are considered sequential downstream questions that should be investigated only if the proximal reproductive predictions receive empirical support. The overall conceptual structure of the Immunological Purity Theory, including the proposed proximal reproductive progression, candidate modifiers, and downstream exploratory extensions, is summarized in **Figure 1**.

**Figure 1:**
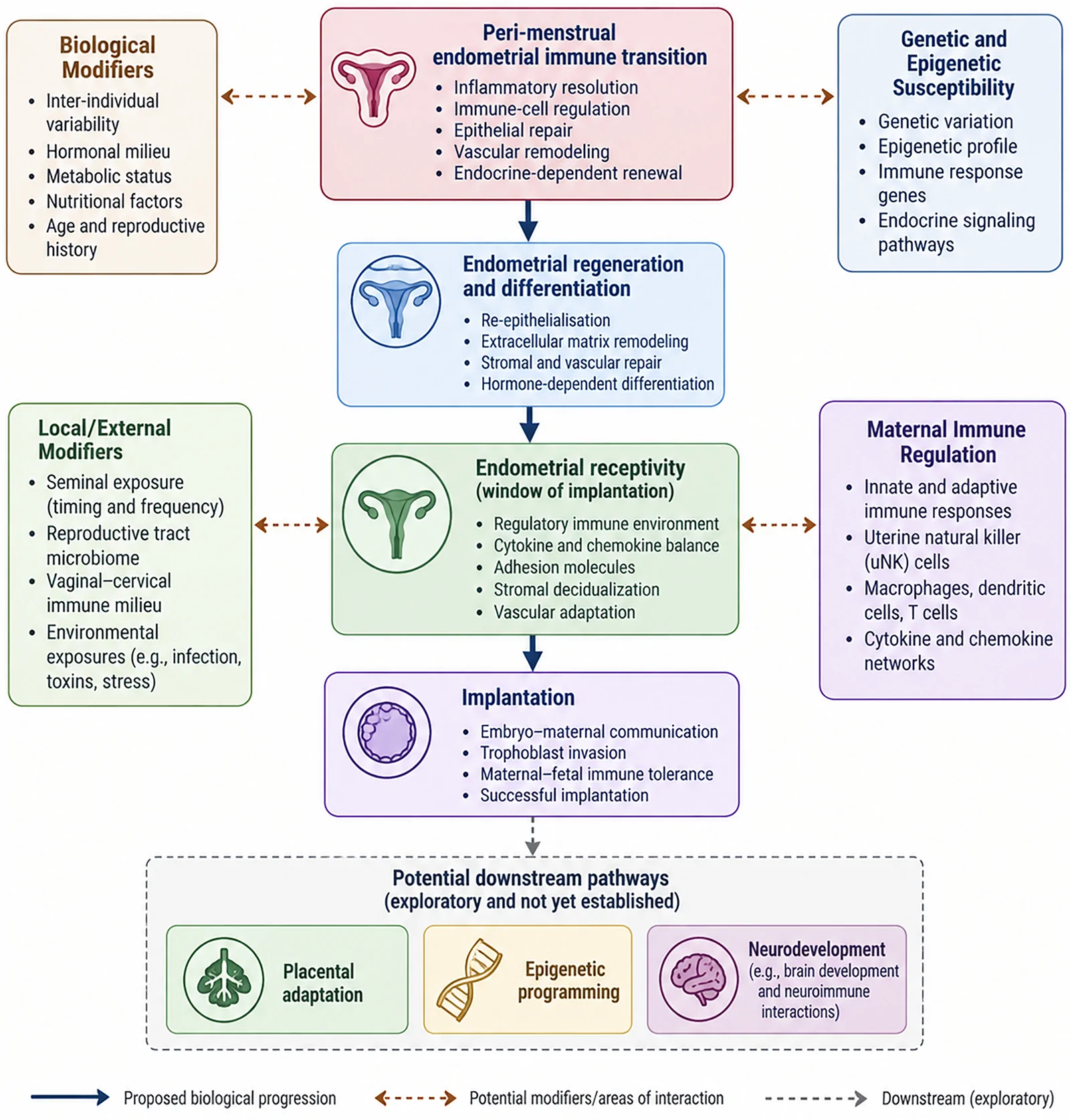
Conceptual framework of the Immunological Purity Theory (IPT). The peri-menstrual endometrial immune transition is proposed as the upstream biological construct, encompassing inflammatory resolution, immune-cell regulation, epithelial repair, vascular remodeling, and endocrine-dependent renewal. The core reproductive hypothesis proposes a prospective relationship from this transition through endometrial regeneration and differentiation to receptivity and implantation within the same menstrual cycle. Biological, genetic, endocrine, environmental, microbial, and immune factors, including seminal exposure, are represented as potential modifiers or interacting influences rather than established components of the core causal pathway. Placental adaptation, epigenetic programming, and neurodevelopment are positioned as progressively downstream exploratory extensions that should be investigated only after validation of the proximal reproductive relationships. Solid arrows represent the proposed proximal biological progression, dashed interaction arrows indicate potential modifiers, and downstream dashed connections denote exploratory relationships. All IPT-specific relationships remain hypothetical and require independent empirical validation; the figure should not be interpreted as an established causal sequence.

Within this framework, the term Immunological Purity Theory is retained as the name of the conceptual model. “Immunological purity” does not denote sterility, immune inactivity, absence of inflammation, or a recognized clinical state. Operationally, the model is evaluated through measurable features of the peri-menstrual endometrial immune transition. The validity of the hypothesis therefore depends not on the terminology itself but on whether its proposed relationships can be prospectively demonstrated, replicated, or falsified.

The objectives of this article are consequently to: (i) critically synthesize evidence concerning menstrual immune resolution, endometrial repair, receptivity, and implantation; (ii) define measurable components of the peri-menstrual endometrial immune transition; (iii) distinguish direct evidence from biological plausibility and theoretical extrapolation; (iv) evaluate candidate modifiers, competing explanations, and methodological limitations; and (v) formulate sequential, falsifiable predictions through which the core reproductive hypothesis and its downstream extensions may be independently tested.

### 1.1 Methodological approach to evidence synthesis and theory development

Consistent with the aims of a Hypothesis and Theory article, this study employs a critical synthesis of multidisciplinary scientific evidence as the analytical foundation for developing and operationalizing a novel, testable, and falsifiable conceptual and theoretical framework. Existing evidence is critically integrated to generate a new interpretation and formulate the Immunological Purity Theory (IPT) as a novel, testable and falsifiable theoretical framework of current knowledge. The purpose of the evidence synthesis is therefore not to provide a conventional narrative, systematic, or scoping review of the literature as an end in itself, but to identify an unresolved biological question, define measurable constructs, specify candidate biological pathways, and derive exploratory and empirically testable questions that can be subjected to prospective testing and falsification.

The analytical approach explicitly distinguishes among three levels of evidence: (i) established component biology supported independently of the IPT; (ii) preliminary or indirect evidence providing biological plausibility or feasibility for specific proposed relationships; and (iii) novel IPT-specific relationships for which direct empirical validation is currently absent. Accordingly, evidence supporting an individual biological domain is not interpreted as evidence supporting the complete proposed pathway. This evidential hierarchy is used throughout the framework to separate established knowledge from theoretical extrapolation and to prevent biological plausibility from being interpreted as causality.

The central construct is operationalized as the peri-menstrual endometrial immune transition rather than as a binary state of “immunological purity.” Candidate measurable domains include cytokine and chemokine signaling, immune-cell composition and phenotype, epithelial repair and regeneration, vascular and extracellular-matrix remodeling, and endocrine responsiveness. The framework further evaluates temporal ordering and the proposed biological bridge from the peri-menstrual immune–repair state through endometrial regeneration and differentiation to later receptivity and implantation within the same menstrual cycle. Potential confounders, candidate mediators, effect modifiers, competing explanations, measurement limitations, and alternative causal structures are considered explicitly.

The theory is structured sequentially to distinguish its core reproductive proposition from its downstream exploratory hypotheses. The core proposition concerns whether measurable variation in the peri-menstrual endometrial immune–repair transition prospectively predicts later endometrial receptivity and implantation. Placental adaptation, epigenetic programming, and neurodevelopment are treated as progressively distal exploratory extensions rather than established consequences of the core hypothesis. Seminal exposure timing is evaluated separately as a candidate effect modifier rather than as a defining causal exposure.

From this conceptual framework, seven sequential and empirically testable predictions are derived. These progress from identification and reproducibility of a peri-menstrual immune–repair phenotype, through prospective prediction of receptivity and implantation, to candidate effect modification, placental consequences, epigenetic mechanisms, and conditional neurodevelopmental extensions. For the principal predictions, the framework specifies primary biological endpoints, directionality where scientifically justified, and explicit falsification criteria. A proposed relationship is therefore considered scientifically informative only insofar as it generates observations capable of supporting, modifying, or refuting the corresponding component of the hypothesis.

Because direct prospective IPT-specific data are not yet available, the framework does not impose arbitrary numerical minimum effect sizes of interest. Instead, initial empirical studies are proposed to estimate effect-size distributions, measurement reliability, within-person and between-person variability, and clinically relevant outcome variation. These data can subsequently inform preregistered minimum effect sizes of biological or clinical interest in independent confirmatory cohorts. Repeated within-person measurement, prospective temporal assessment, preregistration, appropriate control of confounding and multiple testing, independent replication, and transparent reporting of null findings are therefore incorporated as requirements for subsequent empirical validation.

Accordingly, the literature synthesis functions as the analytical foundation for hypothesis generation and theoretical development rather than as the final scientific objective of the article. The principal contribution of the manuscript is the formulation and operationalization of a novel conceptual and theoretical framework with a defined evidential hierarchy, explicit causal alternatives, downstream exploratory hypotheses, seven sequential testable predictions, and prespecified conditions under which individual components can be supported, revised, or falsified through future empirical research.

## 2 Current understanding of endometrial immune transition and receptivity

### 2.1 Menstruation as a coordinated inflammatory and reparative process

Menstruation is a physiological inflammatory and reparative process characterized by tightly coordinated endocrine withdrawal, tissue breakdown, immune-cell recruitment, vascular change, extracellular-matrix remodeling, and rapid initiation of repair. Progesterone withdrawal at the end of the luteal phase promotes inflammatory mediator production, prostaglandin signaling, matrix metalloproteinase activation, leukocyte trafficking, and shedding of the functional endometrial layer (2, 4).

Importantly, tissue breakdown and repair are not strictly sequential events. Re-epithelialisation and vascular restoration begin while menstrual shedding is still occurring, and the human endometrium normally regenerates rapidly without permanent fibrosis (5, 6). Menstruation should therefore not be conceptualized simply as an inflammatory insult followed by passive recovery. Rather, it represents a dynamic transition in which inflammatory activity, tissue clearance, repair, and regeneration overlap temporally.

This distinction is central to the revised Immunological Purity Theory. The framework does not propose that menstruation creates a pathological inflammatory state in healthy women or that menstrual inflammation necessarily persists after bleeding has ceased. Instead, it asks whether the timing, coordination, and biological characteristics of the transition from menstrual inflammation and repair toward subsequent regeneration vary reproducibly between individuals or cycles, and whether any such variation has functional relevance for later endometrial biology.

Accordingly, the proposed exposure is not the presence or absence of inflammation itself. It is the multidimensional peri-menstrual endometrial immune transition, potentially encompassing inflammatory resolution, immune-cell composition and phenotype, epithelial repair, vascular remodeling, extracellular-matrix turnover, and endocrine responsiveness. These domains should initially be characterized as continuous biological variables rather than classified as normal or abnormal before empirical associations with later reproductive outcomes are established.

### 2.2 Resolution, regeneration, and biological variation

Resolution of inflammation is an active biological process involving changes in cytokine signaling, leukocyte trafficking and phenotype, vascular stabilization, epithelial integrity, extracellular-matrix reconstruction, and endocrine responsiveness. In the endometrium, these processes occur within a tissue that subsequently undergoes extensive estrogen-dependent proliferation and progesterone-dependent differentiation during the same menstrual cycle.

Cycle-dependent immune variation is also evident elsewhere in the female reproductive tract. In a systematic review and individual-participant-data meta-analysis, Hughes et al. (32) identified consistent follicular-to-luteal differences across multiple cervicovaginal immune mediators, including chemokines, matrix metalloproteinases, interleukins, and antimicrobial mediators. These findings do not directly characterize the endometrium, but they reinforce the importance of precise cycle staging when interpreting reproductive-tract immune measurements and demonstrate that physiological immune profiles vary systematically across the menstrual cycle.

Interindividual variation in menstrual duration, blood loss, endocrine dynamics, inflammatory mediator expression, leukocyte composition, angiogenesis, and regenerative responses has been described. However, the existence of variation alone does not imply dysfunction. A central methodological requirement for testing the present hypothesis is therefore to distinguish normal physiological variability from an early-cycle phenotype that is reproducible and prospectively informative for later endometrial function.

The first empirical step should be repeated within-person characterization across multiple cycles. A candidate peri-menstrual phenotype would become biologically informative only if it demonstrates reproducibility beyond expected cycle-to-cycle variation and subsequently predicts independently measured features of endometrial differentiation, receptivity, or implantation. Conversely, if early-cycle immune–repair characteristics show poor reproducibility or no prospective relationship with later endometrial function, the core hypothesis would be substantially weakened.

No validated biomarker or composite index currently defines completion of a peri-menstrual endometrial immune transition. The present framework therefore does not propose a diagnostic threshold or clinical classification. Rather, it identifies measurable biological domains from which prospective studies may determine whether a reproducible and functionally relevant phenotype exists.

### 2.3 Endometrial immune cells beyond simple inflammation

The endometrial immune environment cannot be represented by a simple balance between “pro-inflammatory” and “anti-inflammatory” activity. Uterine natural killer cells, macrophages, dendritic cells, regulatory T cells, neutrophils, and other leukocyte populations contribute to tissue breakdown, repair, immune regulation, decidualization, vascular remodeling, and placental development through functions that change across the menstrual cycle (1, 13, 14).

Uterine natural killer cells, for example, differ functionally from classical circulating cytotoxic NK cells and later contribute to vascular remodeling and regulation of trophoblast invasion. Macrophages participate in clearance of cellular debris, tissue repair, angiogenesis, extracellular-matrix remodeling, and immune regulation. Consequently, neither the abundance of a single immune-cell population nor the concentration of an isolated cytokine can adequately define the proposed peri-menstrual immune transition.

Future operationalization should therefore integrate multiple domains, including selected cytokine and chemokine profiles, immune-cell composition and phenotype, epithelial repair markers, vascular and extracellular-matrix remodeling, and endocrine context. The purpose of such multidomain assessment would be to identify reproducible biological patterns rather than to assign an *a priori* binary state of “immunologically pure” or “impure.”

### 2.4 Endometrial receptivity as the primary proximal outcome

Successful implantation requires temporal synchronization between a developmentally competent embryo and a receptive endometrium. Following estrogen-dependent proliferation, progesterone induces extensive epithelial, stromal, vascular, and immune changes that contribute to decidualization and establishment of the mid-secretory receptive state. This process involves coordinated regulation of adhesion molecules, cytokines, chemokines, growth factors, lipid mediators, extracellular vesicles, and immune-cell populations (7, 8, 10, 13).

Receptivity is therefore a multidimensional and temporally restricted phenotype rather than a single molecular marker. Controlled inflammatory signaling participates in implantation, and experimental and clinical literature has highlighted the context-dependent relationship between endometrial inflammation and implantation biology (3, 33).

Endometrial receptivity and implantation are consequently defined as the primary proximal outcomes of the Immunological Purity Theory. Placental, epigenetic, and neurodevelopmental outcomes should not be investigated as primary tests of the framework until the proposed relationship between the peri-menstrual immune transition and these proximal reproductive outcomes has first been demonstrated.

### 2.5 From the peri-menstrual immune transition to later receptivity: the proposed temporal bridge

Menstruation and implantation are temporally separated biological events. Menstruation occurs during the early follicular phase, whereas implantation occurs only after ovulation, within the receptive mid-secretory endometrium. The Immunological Purity Theory therefore does not require persistence of menstrual inflammatory activity until implantation. Instead, it proposes a prospective within-cycle relationship: measurable characteristics of the early immune–repair transition may reflect or predict the subsequent trajectory through which the same endometrial tissue regenerates, proliferates, differentiates, and ultimately acquires a receptive phenotype.

Preliminary human evidence indicates that early-cycle menstrual material can contain biologically informative signals relevant to later reproductive function. In a pilot study of 42 women undergoing frozen embryo transfer, Jain et al. collected menstrual blood directly from the uterine cavity at the beginning of the embryo-transfer cycle and quantified 48 cytokines, chemokines, and growth factors. Several mediators, including IL-6, G-CSF, MCP-1, M-CSF, SDF-1α, IP-10, MIG, and others, differed between women who subsequently achieved pregnancy and those who did not, whereas the measured microbial profiles were not associated with the transfer outcome (11). These findings do not establish causality or demonstrate a specific immune-resolution phenotype, but they provide preliminary human evidence that molecular features measurable during menstruation may carry information associated with a reproductive outcome occurring later in the same cycle.

More recently, Luongo et al. demonstrated that organoids and endometrial stromal cells generated from menstrual blood of healthy fertile donors reproduced key structural and functional features of biopsy-derived endometrial models and responded to hormonal stimulation in ways relevant to decidualization and receptivity (12). This study does not demonstrate that a menstrual immune profile predicts implantation; however, it provides functional evidence that menstrual material can capture biologically informative properties of endometrial tissue and may offer a non-invasive platform for longitudinal investigation of endometrial function.

Together, these studies support the feasibility of testing an early-cycle-to-later-cycle relationship but do not validate the Immunological Purity Theory. The critical missing evidence is a prospective longitudinal study in which peri-menstrual immune–repair characteristics are measured in the same participants and related to subsequent mid-secretory receptivity and implantation using predefined outcomes and repeated-cycle assessment.

The temporal bridge proposed here is therefore explicitly falsifiable. If reproducible peri-menstrual immune–repair phenotypes cannot be identified, or if such phenotypes fail to predict subsequent receptivity or implantation after adjustment for endocrine timing, embryo characteristics, maternal health, and other relevant confounders, the central reproductive component of the hypothesis would not be supported.

## 3 The Immunological Purity Theory: definition and core testable proposition

The Immunological Purity Theory (IPT) is proposed as a conceptual name for a testable reproductive-immunology framework rather than as a description of an established physiological or clinical state. In response to the potential ambiguity of the term *immunological purity*, the biological construct evaluated throughout this article is defined operationally as the peri-menstrual endometrial immune transition. This construct refers to measurable changes accompanying the transition from menstrual-associated inflammatory and reparative activity toward endometrial regeneration and subsequent preparation for receptivity.

The term *purity* does not imply sterility, immune inactivity, complete absence of inflammatory mediators, or a binary distinction between physiologically “pure” and “impure” endometrium. Such interpretations would be inconsistent with contemporary reproductive immunology because regulated immune and inflammatory activity is required for tissue repair, decidualization, implantation, trophoblast invasion, vascular adaptation, and maternal–fetal immune tolerance (1, 3, 13). The term is retained solely as the name of the proposed hypothesis, whereas empirical testing should use measurable biological variables rather than the conceptual label itself.

### 3.1 Core hypothesis

The core reproductive hypothesis is that interindividual and intraindividual variation in the timing, coordination, and biological characteristics of the peri-menstrual endometrial immune–repair transition may prospectively predict subsequent endometrial differentiation, receptivity, and implantation within the same menstrual cycle.

This proposition does not assume that menstrual inflammation persists until implantation. Rather, it predicts that an early-cycle endometrial phenotype may contain information about the subsequent biological trajectory of the same tissue as it undergoes regeneration, estrogen-dependent proliferation, progesterone-dependent differentiation, and acquisition of the receptive mid-secretory phenotype.

The primary directionality proposed by the IPT therefore concerns altered coordination or resolution, rather than simply greater or lesser inflammation. A profile characterized by delayed, prolonged, or otherwise dysregulated coordination among inflammatory resolution, tissue repair, immune-cell regulation, vascular remodeling, and endocrine responsiveness is hypothesized to be associated with a less favorable subsequent receptivity phenotype than a profile in which these processes are appropriately coordinated. This directional prediction remains unvalidated and must be tested prospectively.

Importantly, biological variation should not be interpreted as dysfunction solely because it differs between individuals or cycles. To support the hypothesis, a candidate early-cycle phenotype would need to demonstrate reproducibility, temporal precedence, and prospective association with independently measured receptivity or implantation outcomes beyond variation attributable to cycle timing, endocrine status, maternal health, embryo characteristics, and other relevant confounders.

### 3.2 Candidate biological components

The proposed peri-menstrual endometrial immune transition is multidimensional. Candidate components include cytokine and chemokine signaling, immune-cell composition and phenotype, epithelial repair and regeneration, vascular and extracellular-matrix remodeling, and endocrine responsiveness. These components are proposed as measurable domains for empirical investigation rather than as a validated biomarker panel.

No single cytokine, immune-cell population, histological feature, or molecular marker is currently sufficient to define completion of the proposed transition. Similarly, no evidence currently supports assigning fixed weights to individual biomarkers or calculating a clinical “purity” score. Initial studies should therefore characterize these domains longitudinally and determine empirically which, if any, demonstrate reproducibility and predictive value for subsequent receptivity and implantation.

The framework also does not currently justify a predefined numerical minimum effect size for the proposed relationships. Because direct prospective data are lacking, assigning an arbitrary threshold would create false precision. Exploratory studies should first estimate within-person variability, between-person variability, and effect-size distributions. These data can subsequently inform preregistered minimum effect sizes of biological or clinical interest in independent confirmatory cohorts.

### 3.3 Candidate modifiers: the timing of seminal exposure

The core hypothesis concerns the endogenous peri-menstrual endometrial immune transition. However, this transition occurs within a reproductive environment that may also be influenced by external biological exposures. Seminal exposure is one candidate modifier deserving separate investigation because seminal fluid is biologically active and can induce inflammatory and immune-regulatory responses within the female reproductive tract.

Seminal fluid contains signaling molecules, including transforming growth factor-β and prostaglandins, that can influence epithelial signaling, leukocyte recruitment, regulatory T-cell responses, and reproductive immune adaptation (15, 16). Experimental and human evidence therefore supports the general proposition that seminal exposure can modify the female reproductive immune environment.

Human experimental evidence nevertheless remains limited. In a randomized, double-blind, placebo-controlled study, vaginal seminal-plasma exposure after ovulation was associated with enrichment of endometrial transcriptomic pathways related to immune response, cellular proliferation and movement, implantation, embryo development, and angiogenesis; however, no individual genes remained significantly differentially expressed after correction for multiple testing, and the analyzed sample was small (five seminal-plasma and four placebo participants) (34).

Importantly, existing evidence does not establish that seminal exposure during menstruation or before completion of the proposed peri-menstrual immune transition is harmful, nor does it demonstrate that such exposure impairs subsequent implantation. Seminal-fluid signaling can participate in physiological immune adaptation associated with reproduction (15, 16). However, current evidence does not establish whether the reproductive effects of seminal exposure vary according to the peri-menstrual endometrial immune state at the time of exposure. Therefore, no beneficial or detrimental effect of peri-menstrual seminal exposure should be inferred from the available evidence.

Accordingly, the IPT treats the timing of seminal exposure relative to the peri-menstrual endometrial immune state as a secondary interaction hypothesis, not as an established causal mechanism. The testable question is whether the biological response to seminal exposure differs according to the endometrial immune–repair state at the time of exposure and whether any such interaction predicts subsequent receptivity or implantation. The directional possibility that exposure before completion of an appropriately coordinated immune–repair transition may be associated with a less favorable later receptivity profile is a hypothesis to be tested, not a conclusion of the current evidence.

### 3.4 Boundaries of the hypothesis

The revised framework deliberately separates the core reproductive hypothesis from more distal developmental extensions. Evidence concerning menstrual repair, endometrial immune regulation, receptivity, and implantation provides the immediate biological basis for testing the framework. Placental adaptation, epigenetic programming, and neurodevelopment are downstream hypotheses that become relevant only if the proximal reproductive relationships are first supported.

Thus, the IPT does not currently propose that peri-menstrual immune variation, seminal exposure timing, or their interaction causes autism spectrum disorder. No direct evidence demonstrates such a relationship. Autism and other neurodevelopmental outcomes are retained only as distal exploratory questions motivated by broader evidence concerning developmental programming and maternal immune biology. Each intervening biological transition would require independent empirical validation before causal inference across the complete pathway could be considered.

The hypothesis is therefore falsifiable at multiple levels. Failure to identify reproducible peri-menstrual immune–repair phenotypes would challenge its foundational premise. Identification of such phenotypes without prospective association with receptivity or implantation would weaken the core reproductive hypothesis. Conversely, support for proximal reproductive relationships would justify—but would not itself establish—subsequent investigation of placental, epigenetic, or neurodevelopmental outcomes.

## 4 Relationship to existing theoretical and biological frameworks

The Immunological Purity Theory should be interpreted as an extension of established reproductive and developmental frameworks rather than as evidence against or a replacement for them. Its proposed novelty lies in asking whether measurable variation during the peri-menstrual endometrial immune transition prospectively relates to subsequent receptivity and implantation. Existing frameworks provide biological context for this question, but evidence supporting those frameworks must not be interpreted as direct evidence supporting the IPT.

### 4.1 Reproductive immunology and implantation biology

Reproductive immunology provides the most proximal biological foundation for the hypothesis. Menstruation, endometrial repair, decidualization, implantation, and early placentation involve temporally regulated interactions among endocrine signals, cytokines, chemokines, stromal and epithelial cells, uterine natural killer cells, macrophages, dendritic cells, regulatory T cells, and trophoblast populations (1, 3, 13, 14).

This literature establishes that immune activity is integral to successful reproduction and that the timing and context of inflammatory signaling are biologically important. It also establishes that implantation requires a receptive endometrium generated through coordinated endocrine, cellular, vascular, and immune changes (8, 10).

However, these established principles do not demonstrate the central proposition of the IPT: that measurable variation during the peri-menstrual immune–repair transition predicts the later receptive state within the same cycle. That relationship remains to be tested directly. Accordingly, reproductive immunology provides a mechanistic foundation and defines relevant candidate variables, whereas the proposed early-cycle-to-receptivity relationship constitutes the novel empirical question.

### 4.2 Developmental Origins of Health and Disease

The Developmental Origins of Health and Disease (DOHaD) framework establishes that biological conditions around conception and during early development can influence later phenotypes through interacting metabolic, endocrine, placental, and molecular pathways (23). The peri-conceptional period is therefore recognized as a biologically sensitive interval.

DOHaD supports the general principle that early maternal and embryonic environments can have consequences beyond the immediate reproductive event. It does not, however, establish that peri-menstrual endometrial immune variation is one such exposure. The IPT therefore draws on DOHaD as a developmental precedent rather than as evidence validating its proposed reproductive or neurodevelopmental pathway.

### 4.3 Maternal immune activation

Maternal immune activation (MIA) research provides extensive experimental evidence that sufficiently strong or dysregulated maternal immune stimulation during pregnancy can influence placental signaling, fetal immune development, microglial maturation, neural organization, and offspring behavior (22, 25–27).

Nevertheless, classical MIA paradigms differ fundamentally from the core exposure proposed by the IPT. MIA models commonly involve systemic immune stimulation during established pregnancy, frequently using infection, polyinosinic:polycytidylic acid, lipopolysaccharide, or other inflammatory challenges. By contrast, the IPT concerns physiological or subclinical variation in local endometrial immune–repair biology before implantation.

MIA studies therefore demonstrate that maternal immune signaling **can** influence development under specific conditions; they do not demonstrate that normal peri-menstrual immune variation produces equivalent effects. Human observational associations between maternal inflammatory conditions and neurodevelopment are additionally subject to genetic and familial confounding, exposure heterogeneity, maternal comorbidity, medication use, and environmental and socioeconomic factors (28). MIA is consequently treated as a source of mechanistic plausibility for downstream investigation, not as direct evidence for the IPT.

### 4.4 Placental and epigenetic frameworks

Placental biology provides another potential downstream bridge because the placenta regulates endocrine, vascular, metabolic, and immune communication between mother and fetus (35). Likewise, extensive epigenetic reprogramming occurs during gametogenesis and early embryonic development, including changes in DNA methylation, chromatin organization, and other regulatory processes (24).

These established biological processes make placental adaptation and epigenetic regulation plausible domains for future investigation. However, no direct evidence currently demonstrates that peri-menstrual endometrial immune variation produces persistent placental or embryonic epigenetic alterations. The epigenetic component of the IPT is therefore explicitly classified as a downstream theoretical mechanism rather than an evidence-supported step in the core hypothesis.

Furthermore, detection of an epigenetic difference would not by itself establish mediation or causality. Epigenetic signatures may reflect cell composition, genetic variation, later pregnancy exposures, or other biological processes. Future studies would therefore require temporal measurement, appropriate cell-specific analysis, replication, and functional validation before epigenetic changes could be interpreted as mediators of an early endometrial exposure.

### 4.5 Hierarchy of evidence within the IPT

To prevent conflation of established biology with evidence for the proposed framework, the revised IPT distinguishes three evidential levels:

Established component biology: menstrual inflammation and repair, endometrial regeneration, hormone-dependent differentiation, implantation immunology, placental function, and peri-conceptional developmental sensitivity are supported independently by substantial literature.

Indirect or preliminary evidence relevant to the IPT: early-cycle endometrial or menstrual biological measurements can contain information relevant to later reproductive function, providing feasibility and biological plausibility for prospective investigation.

Direct evidence for the complete IPT pathway: currently absent. No prospective human study has demonstrated the complete sequence from a defined peri-menstrual endometrial immune phenotype through receptivity and implantation to placental, epigenetic, or neurodevelopmental outcomes.

This hierarchy is essential to interpretation of the framework. Evidence that an individual component is biologically established does not constitute evidence that the proposed connections between components are established. The evidence status of the proposed biological transitions is summarized in **Table 1**, while the proposed causal roles of major variables relevant to empirical testing of the IPT are summarized in **Table 2**.

**Table 1:** Evidence status of the biological transitions proposed within the Immunological Purity Theory.

Proposed transition	Mechanistic rationale	Current evidence status	Role in IPT
Peri-menstrual immune–repair transition → later receptivity	Continuous endometrial regeneration, endocrine–immune coordination, tissue remodeling	Preliminary/indirect human evidence; direct prospective validation lacking	Core hypothesis
Receptivity → implantation	Established embryo–endometrial synchronization and implantation biology	Strong, established evidence	Established proximal biology
Implantation → altered maternal–fetal interface	Decidual, trophoblast, immune, vascular and ligand–receptor communication	Strong general mechanistic evidence; not IPT-specific	Secondary mechanistic test
Maternal–fetal interface → placental adaptation	Trophoblast invasion, decidualization, vascular and immune adaptation	Established generally; no direct IPT evidence	Secondary downstream hypothesis
Early reproductive variation → persistent epigenetic programming	Peri-conceptional epigenetic sensitivity	Theoretically plausible; no direct IPT evidence	Hypothetical mechanism
IPT exposure → neurodevelopment/autism	Sequential extrapolation through reproductive and developmental pathways	No direct evidence	Distal exploratory extension
Peri-menstrual activity → later immune priming	Trained-immunity precedent	No direct evidence in this context	Candidate mechanism

Evidence supporting an individual biological domain should not be interpreted as evidence supporting the complete proposed pathway. “IPT-specific evidence” refers to evidence directly linking the peri-menstrual endometrial immune transition to the subsequent outcome.

**Table 2:** Proposed causal roles of major variables relevant to testing the Immunological Purity Theory.

Variable/domain	Potential causal role	Why it matters	Recommended analytical approach
Maternal age, metabolic, endocrine health	Confounder	May influence both immune biology and receptivity	Clinical phenotyping and multivariable adjustment
Maternal/offspring genetics	Confounder or effect modifier	May influence immune regulation and developmental outcomes	Genotyping, family-based approaches where feasible
Cycle timing and hormone exposure	Major confounder	Strongly determines normal endometrial immune and molecular variation	Hormonal confirmation and standardized cycle staging
Embryo competence, genetics	Confounder or interacting determinant	Can influence implantation and maternal signaling	Embryo-quality adjustment; genetic information where appropriate
Endometrial differentiation/decidualization	Potential mediator	May connect early-cycle phenotype with receptivity	Prospective mediation analysis after temporal association is established
Seminal exposure timing	Candidate effect modifier	May alter local immune signaling depending on endometrial state	Predefined interaction analysis
ART procedures	Confounder/effect modifier	Hormonal treatment and procedures may alter endometrial biology	Separate natural-conception and ART cohorts
Placental adaptation	Downstream mediator for developmental outcomes	May transmit implantation-related differences to later pregnancy	Analyze only after proximal reproductive associations are established

Classification as a confounder, mediator, or effect modifier is provisional and must be evaluated according to the specific research question and temporal study design.

## 5 Current knowledge gaps and evidential boundaries

Several unresolved questions define the evidential boundaries of the Immunological Purity Theory. Importantly, these gaps identify what must be tested; their existence should not be interpreted as evidence that the hypothesis is correct.

### 5.1 Is there a reproducible peri-menstrual endometrial immune phenotype?

The first gap concerns the foundational exposure itself. Menstrual and early-follicular biology varies among individuals and across cycles, but no validated multidomain phenotype currently defines the peri-menstrual endometrial immune transition. It remains unknown whether integrated patterns of cytokine and chemokine signaling, immune-cell phenotype, epithelial repair, vascular remodeling, extracellular-matrix turnover, and endocrine context form reproducible within-person profiles across cycles.

Demonstrating variability alone would be insufficient. The foundational premise requires evidence that at least some early-cycle biological characteristics are reproducible beyond expected temporal and technical variation. Failure to demonstrate such reproducibility would substantially weaken the hypothesis before downstream outcomes are considered.

### 5.2 Does an early-cycle phenotype predict later receptivity or implantation?

The second and most important gap concerns the proposed temporal bridge. Menstruation and implantation are separated by substantial endometrial regeneration, proliferation, and differentiation. Preliminary evidence indicates that menstrual material can contain biologically informative endometrial signals, but no adequately powered prospective longitudinal study has established that a defined peri-menstrual immune–repair phenotype predicts mid-secretory receptivity or implantation after adjustment for endocrine timing, maternal health, embryo characteristics, and other confounders.

This relationship represents the primary empirical test of the IPT. If early-cycle phenotypes are measurable but do not prospectively predict later receptivity or implantation, the core reproductive hypothesis would not be supported.

### 5.3 Do candidate modifiers alter the peri-menstrual transition or its reproductive consequences?

A third gap concerns factors that may modify the proposed transition. Endocrine status, uterine contractility, menstrual-flow characteristics, infection, metabolic and inflammatory conditions, psychosocial stress, and other exposures may influence menstrual or endometrial biology. Seminal exposure is also biologically capable of modifying immune signaling in the female reproductive tract, but whether its effects depend on the peri-menstrual endometrial immune state at the time of exposure remains unknown.

These factors should not be incorporated into the core causal pathway without direct evidence. Instead, they should be tested as candidate modifiers or confounders using designs capable of separating their independent and interactive effects.

### 5.4 Are there persistent downstream placental or epigenetic consequences?

Even if an association between peri-menstrual immune transition and implantation were demonstrated, it would not establish persistence into later pregnancy. No longitudinal evidence currently shows that a peri-menstrual endometrial phenotype predicts subsequent placental immune function, vascular development, or persistent embryonic or placental epigenetic signatures.

Placental and epigenetic outcomes therefore represent secondary mechanistic tests that become relevant only after the proximal reproductive relationship has been supported. Their investigation should include temporal sampling and careful control for maternal, embryonic, genetic, metabolic, infectious, and later gestational exposures.

### 5.5 Is there any relationship with neurodevelopment?

The most distal gap concerns neurodevelopment. No direct human or experimental evidence currently demonstrates that peri-menstrual endometrial immune variation predicts autism spectrum disorder or other neurodevelopmental outcomes. Existing MIA, placental, and developmental-programming literatures provide only general biological plausibility for asking whether sufficiently persistent upstream reproductive differences could have later developmental consequences.

Neurodevelopment should therefore not serve as an initial validation endpoint for the IPT. Such investigation would be justified only after reproducible associations have been demonstrated sequentially across the more proximal reproductive and placental stages. Even then, genetic liability, paternal and maternal factors, fetal sex, environmental exposures, pregnancy complications, and postnatal determinants would require rigorous consideration.

### 5.6 Absence of evidence is not evidence for the hypothesis

A central interpretive principle of the revised framework is that an unstudied relationship does not become more likely merely because direct evidence is absent. Statements such as “this has not previously been investigated” identify a research gap; they do not support the proposed mechanism.

The IPT therefore derives its scientific value from generating falsifiable questions at an underexplored intersection of established biological fields, not from treating missing evidence as confirmation. Each proposed transition must acquire independent empirical support before it can contribute to a broader causal model.

## 6 Candidate biological pathways and evidence status

The revised Immunological Purity Theory is organized as a sequence of independently testable biological relationships rather than as a single established causal pathway. This distinction is essential because the strength of evidence differs substantially across the proposed transitions. The most proximal relationship—between the peri-menstrual endometrial immune transition and subsequent receptivity—constitutes the core hypothesis. Placental, epigenetic, and neurodevelopmental pathways represent progressively more distal extensions and should not be interpreted as established consequences of the peri-menstrual state.

### 6.1 Peri-menstrual immune–repair transition to subsequent endometrial receptivity

The first proposed transition concerns whether measurable characteristics of the peri-menstrual endometrial immune–repair state predict the subsequent receptive phenotype within the same cycle. Candidate mediators include cytokine and chemokine signaling, immune-cell composition and phenotype, epithelial restoration, vascular and extracellular-matrix remodeling, and endocrine responsiveness.

The mechanistic rationale derives from the continuity of endometrial tissue remodeling across the menstrual cycle. Menstrual breakdown and repair are followed by estrogen-dependent regeneration and proliferation and, after ovulation, progesterone-dependent differentiation and decidualization-related changes. The proposed relationship therefore does not require persistence of menstrual inflammation. Rather, it predicts that variation in the timing or coordination of early immune–repair processes may be associated with the later developmental trajectory of the endometrium.

Preliminary human studies demonstrate that menstrual material can contain biologically informative signals relevant to later endometrial function (11, 12). However, these findings do not establish that an early immune–repair phenotype causes or predicts receptivity. This transition should therefore be classified as biologically plausible and preliminarily supported, but not directly validated.

### 6.2 Receptivity to implantation: the best-established proximal transition

Among the relationships incorporated into the framework, the link between an appropriately receptive endometrium and implantation is the most firmly established. Implantation requires temporal synchronization between embryo competence and a receptive maternal endometrium involving epithelial adhesion, stromal differentiation, immune regulation, vascular adaptation, cytokine and growth-factor signaling, and embryo–maternal communication (7, 8, 10, 13).

This established biology does not demonstrate that peri-menstrual variation determines receptivity. Instead, it identifies the proximal biological outcome against which the core IPT prediction should be tested. Prospective studies should therefore measure receptivity independently rather than infer it solely from an early-cycle biomarker profile.

### 6.3 Implantation and the maternal–fetal interface

If an association between early-cycle biology and implantation is established, the next mechanistic question concerns whether implantation-associated differences correspond to altered decidual, trophoblast, immune, or vascular interactions at the maternal–fetal interface.

Contemporary single-cell research demonstrates that the maternal–fetal interface is characterized by extensive communication among trophoblast, decidual stromal, epithelial, vascular, and immune-cell populations. Ligand–receptor signaling and cell–cell communication contribute to immune tolerance, tissue remodeling, placental development, and pregnancy maintenance (36).

Recent experimental evidence further demonstrates that inflammatory perturbation can alter these communication networks. In a 2025 single-cell RNA-sequencing study, Lund et al. reported that lipopolysaccharide-induced inflammation altered cellular composition and disrupted multiple signaling pathways involved in embryo–maternal, stromal–epithelial, and stromal–immune communication at the maternal–fetal interface. Pro-inflammatory mediators including CCL2, TNF-α, and IL-1β were increased, while predicted communication through several implantation-relevant pathways was altered (37).

These findings demonstrate that inflammatory perturbation can influence maternal–fetal cellular communication under experimental conditions. They do not demonstrate that physiological peri-menstrual immune variation produces comparable effects. This transition is therefore classified as mechanistically supported in broader implantation biology but untested in relation to the IPT exposure.

### 6.4 Placental adaptation as a secondary downstream hypothesis

The placenta regulates endocrine signaling, nutrient and gas exchange, vascular adaptation, immune tolerance, and communication between maternal and fetal compartments (35). Placental function may also influence fetal brain development through endocrine, metabolic, vascular, and signaling pathways, providing a broader biological rationale for considering the placenta in developmental models (38). Altered implantation and decidualization can plausibly influence early placental development because trophoblast invasion and maternal vascular remodeling begin at the implantation interface.

Nevertheless, no evidence currently demonstrates that a peri-menstrual endometrial immune phenotype produces persistent placental alterations. Placental adaptation is therefore not part of the primary validation criterion for the IPT. It represents a secondary downstream hypothesis that should be investigated only if the preceding relationships between early-cycle phenotype, receptivity, and implantation are supported.

Future studies examining this transition should prioritize proximal placental outcomes, including trophoblast invasion, decidual immune composition, vascular remodeling, placental cytokine signaling, and cell–cell communication, before considering more distal developmental consequences.

### 6.5 Epigenetic programming as a hypothetical downstream mechanism

Extensive epigenetic reprogramming occurs during gametogenesis, preimplantation development, implantation, and early embryogenesis, involving DNA methylation remodeling, chromatin reorganization, histone modification, and other regulatory processes (24). These processes provide a general biological basis for investigating whether sufficiently persistent changes in the peri-conceptional environment could influence developmental programming.

However, no direct evidence currently demonstrates that peri-menstrual endometrial immune variation induces persistent embryonic or placental epigenetic alterations. The presence of an epigenetic difference would also not, by itself, demonstrate mediation or causality because such differences may reflect cell composition, genetic variation, maternal exposures later in pregnancy, or other biological processes.

Epigenetic programming is therefore classified in the IPT as a hypothetical downstream mechanism, not an evidence-supported link. Investigation of this mechanism would require temporal sampling, cell-specific analysis, replication, and functional validation after the proximal reproductive relationships have first been established.

### 6.6 Neurodevelopment as a distal exploratory extension

Maternal immune activation studies demonstrate that substantial immune perturbations during established pregnancy can alter placental signaling, fetal immune development, microglial maturation, neural organization, and offspring behavior in experimental models (22, 25–27). Microglia themselves have developmentally dynamic functions extending beyond classical inflammatory responses to neural development, circuit remodeling, and central nervous system homeostasis (39). Human studies also report associations between several maternal inflammatory conditions and neurodevelopmental outcomes, although causal interpretation remains limited by genetic, familial, clinical, environmental, and socioeconomic confounding (28).

Neuroinflammatory and glial findings have also been reported in autism-related human and experimental literature, including altered microglial and astrocytic phenotypes and immune signaling; however, whether such findings represent causal mechanisms, downstream consequences, or heterogeneous correlates of autism remains unresolved (40).

These findings provide a biological rationale for considering neurodevelopment as a potential downstream research domain. They do not provide evidence that peri-menstrual immune variation influences neurodevelopment. The magnitude, timing, and biological context of classical maternal immune activation differ substantially from the physiological or subclinical endometrial variation proposed by the IPT.

Autism spectrum disorder is therefore retained only as a distal exploratory outcome rather than as a primary prediction of the framework. Autism is heterogeneous and strongly influenced by genetic architecture (29–31). No direct evidence currently links the proposed peri-menstrual endometrial phenotype to autism susceptibility, presentation, or severity.

Investigation of neurodevelopment would become scientifically justified only after reproducible evidence supports the preceding sequence of proximal reproductive and placental relationships. Earlier outcomes—including implantation, decidualization, trophoblast behavior, placental development, and neonatal immune phenotypes—should take precedence over autism diagnosis in the empirical testing hierarchy.

### 6.7 Immune priming: a candidate mechanism requiring direct validation

Innate immune cells can undergo functional reprogramming following previous inflammatory stimulation, commonly described as trained immunity, through metabolic and epigenetic mechanisms that influence subsequent responses (41). This phenomenon provides a conceptual precedent for biological memory within innate immune systems.

However, there is currently no direct evidence that normal peri-menstrual immune activity generates a persistent trained-immunity state that subsequently alters implantation. The IPT therefore does not assume immune priming as an established intermediate mechanism. It is retained only as a candidate experimental question: whether repeated or temporally altered peri-menstrual inflammatory exposures produce measurable changes in later endometrial immune responsiveness.

Failure to demonstrate such persistence would remove immune priming from the mechanistic framework without invalidating the more proximal hypothesis concerning early-cycle phenotype and later receptivity.

## 7 Alternative explanations and causal structure

Any association between the peri-menstrual endometrial immune transition and subsequent reproductive outcomes could arise through mechanisms other than those proposed by the Immunological Purity Theory. The revised framework therefore distinguishes confounders, potential mediators, and effect modifiers. This distinction is necessary to avoid interpreting association as evidence of a direct causal pathway.

### 7.1 Potential confounders

Confounders are factors that may influence both the proposed peri-menstrual phenotype and subsequent reproductive or developmental outcomes without lying on the causal pathway proposed by the IPT. Important candidates include maternal age, genetic background, metabolic and endocrine disorders, obesity, autoimmune disease, infection, medication exposure, smoking, nutrition, psychosocial stress, environmental exposures, infertility diagnosis, assisted-reproduction procedures, and socioeconomic factors.

Embryonic characteristics require particular consideration. Embryo competence and genetic variation can influence implantation signaling and maternal endometrial responses. Consequently, an observed association between an endometrial immune profile and implantation outcome could partly reflect embryo-driven signaling rather than a unidirectional maternal effect. Future studies should therefore account for embryo quality and, where ethically and methodologically appropriate, embryonic genetic characteristics.

Normal cycle timing is another critical source of confounding. Cytokine expression, immune-cell populations, endometrial thickness, vascular characteristics, and gene expression change physiologically across the cycle. Precise hormonal and temporal staging is therefore essential to distinguish a candidate IPT phenotype from normal differences caused by sampling at biologically different cycle stages.

### 7.2 Potential mediators

If the core hypothesis is supported, candidate mediators are biological processes through which an early-cycle phenotype could influence a later reproductive outcome. These may include altered endometrial regeneration, progesterone responsiveness, stromal differentiation, decidualization, epithelial receptivity, vascular remodeling, or embryo–endometrial signaling.

Importantly, these variables should not automatically be classified as mediators merely because they occur between menstruation and implantation. Mediation requires evidence of temporal ordering and statistical or experimental support showing that the proposed intermediate process accounts for part of the relationship between the early exposure and later outcome.

Placental adaptation and epigenetic programming should not be treated as mediators of the **core** reproductive hypothesis because they occur downstream of implantation. They become candidate mediators only in separate analyses addressing more distal developmental outcomes.

### 7.3 Potential effect modifiers

Effect modifiers are factors that may change the magnitude or direction of the association between the peri-menstrual immune transition and later reproductive outcomes. Candidate modifiers include endocrine status, maternal metabolic or inflammatory state, uterine contractility, menstrual-flow characteristics, embryo characteristics, and reproductive-tract exposures.

Seminal exposure is included in this category. Seminal fluid can induce inflammatory and regulatory immune responses in the female reproductive tract (15, 16). However, whether the effect of seminal exposure differs according to the peri-menstrual endometrial immune state at the time of exposure has not been directly tested.

The IPT therefore generates a secondary interaction question: does the biological effect of seminal exposure depend on the endometrial immune–repair state at the time of exposure? The directional prediction that exposure occurring before completion of an appropriately coordinated peri-menstrual immune–repair transition could be associated with a less favorable later receptivity profile remains hypothetical and requires direct prospective testing. Existing evidence should not be interpreted as demonstrating that menstrual or peri-menstrual seminal exposure is harmful.

### 7.4 Competing explanations

Several competing explanations could account for future associations without supporting the IPT. First, measurable peri-menstrual variation may represent normal physiological diversity without functional consequences. Second, endocrine timing may explain both early immune profiles and later receptivity. Third, embryo-derived signals may influence maternal biology in ways that create apparent maternal associations. Fourth, shared genetic or metabolic determinants may influence both maternal immune regulation and reproductive or developmental outcomes. Finally, the endometrium may possess sufficient biological resilience to compensate for modest early-cycle variation, resulting in no measurable effect on implantation.

These alternatives are not secondary considerations; they constitute explicit competing models against which the IPT must be tested. Evidence supporting one of these explanations more strongly than the proposed pathway should lead to modification or rejection of the relevant component of the hypothesis.

## 8 Critical evaluation of the current evidence

The current evidence base should be interpreted according to the biological question it can legitimately address. Substantial literature establishes the physiology of menstrual inflammation and repair, the immunology of endometrial receptivity and implantation, and the developmental importance of maternal–fetal communication. However, evidence for these individual biological domains does not establish the connections proposed by the IPT.

### 8.1 What is currently supported

Several principles relevant to the framework are well supported:

1. menstruation involves coordinated inflammatory, tissue-remodeling, and reparative processes;
2. the endometrium undergoes extensive regeneration and endocrine-dependent differentiation across the cycle;
3. implantation requires coordinated embryo–endometrial, immune, vascular, and molecular communication;
4. menstrual material can contain biologically informative endometrial signals and cells;
5. seminal fluid can modify immune signaling within the female reproductive tract; and
6. maternal–fetal and placental communication is sensitive to inflammatory perturbation.

These findings establish biological plausibility and experimental tractability for components of the framework. They do not demonstrate that the complete IPT pathway exists.

### 8.2 What remains unsupported

No study has yet demonstrated that:

- a validated peri-menstrual endometrial immune–repair phenotype exists;
- such a phenotype reproducibly predicts mid-secretory receptivity in healthy women;
- the timing of seminal exposure relative to this phenotype modifies subsequent receptivity or implantation;
- a peri-menstrual phenotype produces persistent placental or epigenetic alterations; or
- peri-menstrual endometrial immune variation predicts autism or other neurodevelopmental outcomes.

These are not minor missing details. They represent the principal empirical transitions on which the hypothesis depends.

### 8.3 Strength of inference

The strongest current inference is therefore that the proposed early-cycle-to-later-receptivity relationship is biologically plausible and experimentally testable. Evidence is insufficient to characterize it as established, causal, clinically predictive, or clinically actionable.

Likewise, evidence concerning seminal-fluid immunobiology supports investigation of exposure timing as a potential modifier but does not establish that exposure during a particular menstrual interval is beneficial or harmful. Evidence concerning maternal immune activation, placental biology, and epigenetic programming supports downstream research questions but cannot substitute for direct evidence linking the peri-menstrual phenotype to those outcomes.

The revised framework therefore adopts a deliberately sequential evidential standard: each proposed transition must be demonstrated independently before evidence from one biological domain is used to justify inference about the next.

## 9 Testable predictions and falsification criteria

The scientific value of the Immunological Purity Theory depends on its ability to generate predictions that can be independently tested and potentially falsified. The revised framework therefore prioritizes proximal reproductive predictions before placental or neurodevelopmental extensions. Each prediction specifies the proposed exposure, primary outcome, expected direction where justified, and the evidence that would weaken or refute the corresponding component of the hypothesis.

### 9.1 Prediction 1: a reproducible peri-menstrual endometrial immune–repair phenotype can be identified

Prediction. If the foundational premise of the IPT is correct, multidomain assessment during the peri-menstrual transition should identify reproducible patterns of endometrial immune–repair biology that cannot be explained solely by sampling time or technical variability.

Candidate domains include cytokine and chemokine signaling, immune-cell composition and phenotype, epithelial repair markers, vascular and extracellular-matrix remodeling, and endocrine context. Initial studies should measure these domains repeatedly across at least two or more cycles within the same participants to estimate both within-person and between-person variability.

Primary endpoint. The primary endpoint should be the reproducibility of a prespecified multidomain biological profile or its component measures across repeated cycles after standardized hormonal and temporal staging.

Directionality. No assumption should be made that globally “higher” or “lower” inflammation represents a favorable phenotype. The directional prediction concerns the timing and coordination of inflammatory resolution and tissue repair rather than absolute inflammatory magnitude.

Falsification criterion. The foundational premise would be weakened if candidate profiles show poor within-person reproducibility after adjustment for cycle stage and technical variability, or if apparent biological patterns are explained predominantly by sampling timing rather than stable or systematically varying endometrial characteristics.

### 9.2 Prediction 2: the peri-menstrual phenotype prospectively predicts later receptivity

Prediction. If early-cycle endometrial biology is functionally relevant, prespecified peri-menstrual immune–repair characteristics should prospectively associate with independently measured features of mid-secretory endometrial receptivity within the same cycle.

Primary endpoints. Outcomes should include validated or independently quantified features of endometrial differentiation and receptivity, potentially incorporating histological, molecular, immune, vascular, and endocrine measures rather than reliance on a single commercial receptivity test.

Directionality. The IPT predicts that profiles characterized by delayed, prolonged, or poorly coordinated resolution of menstrual-associated inflammatory and reparative activity will be associated with a less favorable subsequent receptivity phenotype than profiles characterized by appropriately coordinated transition. This directional prediction requires empirical validation and should not be interpreted as evidence that any isolated inflammatory marker is intrinsically detrimental.

Falsification criterion. The core hypothesis would be substantially weakened if reproducible early-cycle profiles fail to predict later receptivity after adjustment for hormonal timing, maternal health, cycle characteristics, and other predefined confounders.

### 9.3 Prediction 3: early-cycle phenotype predicts implantation-related outcomes

Prediction. If the relationship between early-cycle biology and receptivity is biologically meaningful, the relevant peri-menstrual phenotype should also prospectively associate with implantation-related outcomes.

Primary endpoints. Initial studies should prioritize proximal outcomes such as implantation, biochemical pregnancy, clinical pregnancy, decidualization-related markers, and trophoblast–endometrial interaction. Live birth may be evaluated as a clinically important secondary outcome but is influenced by numerous events occurring after implantation and should not serve as the sole test of the early-cycle hypothesis.

Directionality. A less favorable receptivity-associated early phenotype is predicted to correspond to reduced implantation success or altered implantation-related biological markers, provided embryo competence and other major determinants are adequately controlled.

Falsification criterion. If a peri-menstrual phenotype predicts molecular receptivity measures but shows no reproducible association with implantation-related outcomes in adequately powered and replicated studies, its proposed reproductive significance would require substantial revision.

### 9.4 Prediction 4: seminal exposure timing may modify the association between peri-menstrual immune state and later receptivity

Prediction. Seminal exposure is hypothesized to function as a candidate effect modifier rather than a defining component of the core IPT. If the local biological response to seminal fluid depends on the endometrial immune–repair state at the time of exposure, the relationship between seminal exposure and subsequent receptivity should differ according to objectively measured peri-menstrual endometrial state.

Primary endpoint. The primary analysis should test a prespecified statistical interaction between objectively characterized endometrial state and seminal exposure timing in relation to subsequent receptivity or implantation-related outcomes.

Directionality. A secondary directional prediction of the IPT is that seminal exposure occurring before completion of an appropriately coordinated peri-menstrual immune–repair transition may be associated with a less favorable subsequent receptivity profile than exposure occurring after restoration of endometrial homeostasis. Existing evidence does not establish this direction, and the prediction is explicitly presented for prospective testing.

Falsification criterion. This secondary prediction would not be supported if the effect of seminal exposure does not differ by objectively measured endometrial state, or if earlier exposure is associated with equivalent or more favorable receptivity after appropriate control for confounding.

Importantly, failure of Prediction 4 would not by itself invalidate Predictions 1–3 because seminal exposure timing is proposed as an effect modifier rather than as the foundational exposure of the core hypothesis.

### 9.5 Prediction 5: proximal reproductive differences precede any persistent placental consequences

Prediction. If validated differences in peri-menstrual phenotype and implantation biology persist beyond implantation, corresponding differences may subsequently be detectable in placental or maternal–fetal interface biology.

Primary endpoints. Candidate outcomes include decidual immune composition, trophoblast invasion, vascular remodeling, placental cytokine signaling, and cell–cell communication at the maternal–fetal interface.

Directionality. No universal directional prediction is currently justified for all placental measures because the expected effect will depend on the specific proximal reproductive phenotype identified in earlier studies.

Falsification criterion. If robust early-cycle and implantation associations are established but no corresponding placental differences are observed across adequately powered longitudinal studies, the placental extension of the IPT would be weakened without necessarily invalidating the core reproductive hypothesis.

### 9.6 Prediction 6: epigenetic differences, if present, must follow rather than substitute for proximal validation

Prediction. Persistent epigenetic differences should be investigated only after a reproducible early-cycle phenotype and its association with implantation or placental biology have been established.

Primary endpoints. Candidate measures may include cell-specific DNA methylation, chromatin accessibility, histone-related regulatory signatures, or other validated epigenomic measures collected at biologically appropriate time points.

Falsification criterion. Absence of reproducible epigenetic differences would weaken the epigenetic extension but would not invalidate the core reproductive hypothesis. Conversely, detection of an epigenetic association without prior demonstration of the proximal reproductive pathway would not constitute evidence supporting the complete IPT.

### 9.7 Prediction 7: neurodevelopmental associations are conditional distal predictions

Prediction. Neurodevelopmental outcomes should be investigated only if preceding reproductive and placental predictions are supported. No direct association with autism spectrum disorder is assumed.

Initial longitudinal research should prioritize dimensional and biologically proximal developmental measures before categorical diagnoses. Any later investigation of autism should account for genetic liability, maternal and paternal factors, fetal sex, pregnancy complications, environmental exposures, socioeconomic variables, and postnatal determinants.

Falsification criterion. Absence of a neurodevelopmental association would refute the distal developmental extension but would not invalidate an independently supported reproductive component of the IPT.

### 9.8 Effect-size thresholds and replication requirements

The present evidence base does not permit a scientifically defensible numerical minimum effect size for the novel IPT-specific relationships. Assigning a fixed threshold at this stage would therefore create unjustified precision. Initial discovery studies should estimate effect-size distributions, measurement reliability, within-person variability, and clinically relevant outcome variation.

Subsequent confirmatory studies should preregister minimum effect sizes of interest based on those empirical distributions, use independent replication cohorts, and report confidence intervals alongside statistical significance. Evidence supporting the IPT should require replication and effect-size consistency, not isolated nominally significant associations.

High-dimensional analyses involving cytokines, transcriptomics, microbiome, metabolomics, or epigenomics should additionally use prespecified analytical pipelines and appropriate correction for multiple testing.

## 10 Future directions and sequential research priorities

Empirical evaluation of the Immunological Purity Theory should proceed sequentially. The framework should not initially be tested by searching for direct associations between menstrual characteristics and autism or other distal developmental outcomes. Instead, each proposed biological transition should be evaluated independently, beginning with the existence and reproducibility of the peri-menstrual endometrial immune phenotype and progressing to receptivity, implantation, placental biology, and only subsequently to developmental outcomes.

This staged approach is necessary because failure at an earlier step would substantially alter the rationale for investigating more distal components of the framework.

### 10.1 Phase I: define the peri-menstrual endometrial immune–repair phenotype

The first priority is to determine whether reproducible biological variation can be identified during the peri-menstrual endometrial immune transition in healthy reproductive-age women.

Participants should ideally be studied longitudinally across multiple menstrual cycles rather than sampled once cross-sectionally. Precise cycle staging should incorporate menstrual timing and objective endocrine measurements where feasible. Candidate measures may include:

- cytokine and chemokine profiles;
- immune-cell composition and phenotype;
- epithelial repair and regeneration markers;
- vascular and extracellular-matrix remodeling markers;
- endocrine measurements;
- menstrual-flow characteristics; and
- non-invasive or minimally invasive measures capable of reflecting endometrial biology.

Menstrual blood is particularly relevant as a potential research substrate because it permits repeated sampling without endometrial biopsy and has been shown to contain biologically informative endometrial cells and molecular signals (11, 12). However, menstrual-blood measurements should not automatically be assumed to represent the complete tissue-level endometrial state. Validation against appropriately timed tissue or other reference measures will be necessary.

The primary objective of Phase I is phenotype characterization and reproducibility, not identification of disease. Initial analyses should quantify within-person variability, between-person variability, measurement reliability, and the contribution of cycle timing and endocrine state.

A failure to identify reproducible biological structure beyond expected cycle and technical variation would substantially weaken the foundational premise of the IPT.

### 10.2 Phase II: test the temporal bridge to receptivity

If reproducible early-cycle phenotypes are identified, the next priority is to determine whether they prospectively predict subsequent endometrial differentiation and receptivity within the same cycle.

This phase requires paired early- and later-cycle assessment in the same participants. Peri-menstrual measurements should be collected before the receptive phase, followed by independent mid-secretory assessment of endometrial function.

Outcomes should not rely exclusively on a single commercial receptivity assay. A multimodal approach may incorporate appropriately validated molecular, immune, vascular, histological, endocrine, or functional measures of endometrial differentiation and receptivity.

The key analysis is prospective: does the early-cycle phenotype explain later receptive-state variation beyond normal cycle timing, endocrine status, maternal characteristics, and technical variation?

This phase directly tests the principal temporal bridge challenged in the current literature and should precede investigation of neurodevelopmental outcomes.

#### 10.2.1 Study-design and sample-size considerations for Predictions 1 and 2

Prediction 1 would be most appropriately evaluated using a prospective longitudinal repeated-measures design in which participants are characterized across at least two menstrual cycles, permitting estimation of within-person and between-person variability, measurement reliability, and phenotype reproducibility. Prediction 2 would require paired prospective assessment within the same menstrual cycle, with prespecified peri-menstrual immune–repair measurements obtained before an independently assessed mid-secretory receptivity outcome. Where feasible, repeated-cycle validation should be incorporated to determine whether observed early-to-later-cycle relationships are reproducible rather than cycle-specific.

A definitive sample size cannot currently be specified because no validated IPT-specific effect-size estimate or reproducibility parameter is yet available. For Prediction 1, initial pilot studies should estimate intraclass correlation coefficients, within-person and between-person variance components, assay reliability, and cycle-to-cycle variability. For Prediction 2, pilot data should estimate the magnitude and uncertainty of the prospective association between the prespecified early-cycle phenotype and later receptivity, together with outcome variance, covariate structure, and expected attrition. These empirically derived parameters should then be used in formal power calculations for subsequent preregistered confirmatory studies.

Accordingly, initial studies should be explicitly designated as parameter-estimation and feasibility studies rather than as definitive hypothesis-confirmation studies. Subsequent confirmatory cohorts should prespecify the primary exposure and outcome, target statistical power, significance level, minimum effect size of biological or clinical interest, expected attrition, and—where repeated measurements are used—the within-participant correlation structure. This staged approach avoids imposing an arbitrary sample-size requirement before the key variance and effect-size parameters of the IPT have been empirically characterized.

### 10.3 Phase III: test implantation and the role of candidate modifiers

Only after an association with receptivity is demonstrated should studies evaluate implantation-related outcomes. Prospective natural-conception cohorts and assisted-reproduction cohorts may provide complementary information but should be analyzed separately because ovarian stimulation, exogenous hormones, embryo selection, and transfer procedures can alter endometrial biology.

Primary outcomes may include implantation, biochemical pregnancy, clinical pregnancy, decidualization-related measures, and trophoblast–endometrial interaction. Embryo quality and relevant maternal factors must be accounted for because implantation is determined jointly by maternal and embryonic biology.

This phase should also test prespecified candidate effect modifiers, including seminal exposure timing. Rather than classifying participants solely according to intercourse dates, studies should relate exposure timing to an objectively measured peri-menstrual endometrial state.

A suitable design would test whether the association between seminal exposure and later receptivity or implantation differs according to the biological state of the endometrium at the time of exposure. This would permit direct evaluation of the secondary IPT prediction without assuming in advance that peri-menstrual exposure is harmful.

Such studies should distinguish seminal exposure from sexual activity more broadly because the proposed biological mechanism concerns exposure of the female reproductive tract to seminal fluid rather than sexual behavior itself.

### 10.4 Phase IV: investigate maternal–fetal interface and placental consequences

Placental investigation should follow—not precede—validation of the proximal reproductive pathway. If reproducible early-cycle phenotypes predict receptivity or implantation, longitudinal studies can then determine whether these associations correspond to measurable differences at the maternal–fetal interface.

Candidate outcomes include:

- decidual immune-cell composition;
- trophoblast invasion and differentiation;
- vascular remodeling;
- placental cytokine and chemokine signaling;
- ligand–receptor and cell–cell communication; and
- placental growth and functional measures.

Recent single-cell studies provide increasingly detailed tools for investigating cellular communication at the maternal–fetal interface (36), while experimental evidence demonstrates that inflammatory perturbation can alter these networks (37). These approaches may therefore be useful for mechanistic investigation if the earlier IPT predictions are supported.

### 10.5 Phase V: evaluate developmental programming before categorical neurodevelopmental diagnoses

Developmental follow-up should initially prioritize biologically proximal outcomes rather than autism diagnosis. Candidate measures may include neonatal immune profiles, placental molecular signatures, birth outcomes, early developmental trajectories, and validated dimensional measures of neurodevelopment.

Epigenetic analyses may be incorporated at this stage if earlier phases demonstrate a reproducible reproductive pathway. Such analyses should use cell-specific or appropriately adjusted approaches and should not interpret molecular association as mediation without temporal and functional evidence.

Only after reproducible evidence links the peri-menstrual phenotype sequentially to reproductive and developmental intermediates should long-term studies examine categorical neurodevelopmental outcomes, including autism spectrum disorder.

Any such studies would require sufficiently large cohorts and rigorous adjustment for genetic liability, maternal and paternal characteristics, fetal sex, pregnancy complications, infection, medication exposure, environmental factors, socioeconomic conditions, and postnatal influences.

### 10.6 Replication, preregistration, and analytical rigor

Given the multidimensional nature of the proposed framework, false-positive findings represent a major methodological risk. Future confirmatory studies should therefore preregister primary exposures, outcomes, covariates, interaction tests, minimum effect sizes of interest, and statistical analysis plans.

Discovery and confirmation should be separated wherever feasible. Biomarker selection or phenotype derivation performed in one cohort should be tested in an independent replication cohort. High-dimensional cytokine, transcriptomic, microbiome, metabolomic, and epigenomic analyses should apply appropriate multiple-testing correction and report effect sizes and confidence intervals rather than relying solely on nominal statistical significance.

Repeated within-person sampling is particularly important because a single menstrual cycle cannot establish whether an observed phenotype represents a stable individual characteristic, a transient cycle-specific state, or technical variation.

### 10.7 Translational boundaries

The present framework does not justify clinical recommendations concerning timing of intercourse, conception, menstrual practices, fertility treatment, or prevention of neurodevelopmental conditions.

In particular, the secondary hypothesis concerning seminal exposure timing should not be interpreted as evidence that intercourse during menstruation or the peri-menstrual interval is harmful. No direct evidence currently establishes such a conclusion.

Clinical translation would require replicated prospective evidence demonstrating a biologically meaningful exposure, a reproducible effect on clinically relevant reproductive outcomes, and a favorable balance of benefits and harms from any proposed intervention.

Until such evidence exists, the IPT should remain a research framework rather than a basis for reproductive or neurodevelopmental guidance.

## 11 Critical appraisal of the evidence base

The evidence relevant to the Immunological Purity Theory spans menstrual physiology, endometrial immunology, implantation biology, assisted reproduction, reproductive-tract microbiology, seminal-fluid immunobiology, placental biology, maternal immune activation, developmental programming, and neurodevelopment. This breadth provides multiple mechanistic precedents but also creates a major inferential challenge: evidence derived from one biological domain cannot automatically validate a proposed transition into another.

The strongest evidence relevant to the IPT concerns established component biology, particularly menstrual inflammation and repair, hormone-dependent endometrial differentiation, and the immunological requirements of implantation. The weakest evidence concerns the novel connections proposed by the framework, especially the prospective relationship between peri-menstrual immune state and later receptivity, the interaction with seminal exposure timing, and the downstream extension toward placental, epigenetic, and neurodevelopmental outcomes.

### 11.1 Endometrial receptivity: biological validity versus measurement uncertainty

Endometrial receptivity is biologically well established but difficult to reduce to a single clinical measurement. Implantation requires synchronized endocrine, epithelial, stromal, immune, and vascular changes, yet no individual biomarker universally defines the receptive state (8, 10, 13).

This distinction is important for testing the IPT. A statistically significant association between an early-cycle phenotype and one receptivity marker would not necessarily establish a biologically meaningful effect on receptivity as a whole.

Evidence from personalized receptivity testing illustrates this problem. Glujovsky et al. (42), in a systematic review and meta-analysis of endometrial receptivity test-guided personalized embryo transfer, found no clear improvement in live birth or clinical pregnancy among women without recurrent implantation failure, while evidence in recurrent implantation failure remained uncertain. Heterogeneity in tests, populations, protocols, and study quality limited interpretation.

More recently, Teh et al. (43) investigated cycle-to-cycle variation in paired mid-luteal endometrial biopsies and demonstrated substantial molecular variation across cycles. Although the study was small and focused on mid-luteal gene expression rather than peri-menstrual biology, it reinforces the need for repeated within-person sampling before treating a single-cycle molecular profile as a stable individual characteristic.

Future IPT studies should therefore avoid defining receptivity through a single commercial test or isolated molecular marker. Multimodal assessment and repeated-cycle designs are more appropriate for determining whether an early-cycle phenotype predicts a later functional state.

### 11.2 Reproductive-tract microbiome: potentially relevant but methodologically vulnerable

Reproductive-tract microbial ecology remains one of the most methodologically contested domains relevant to implantation. Associations have been reported between microbial profiles, local inflammatory signaling, infertility, implantation failure, and pregnancy loss (17, 19, 20, 44).

However, the endometrium is a low-biomass environment. Microbial DNA detected in endometrial samples may derive from true biological material, transient microbial passage, contamination introduced during transcervical sampling, laboratory reagents, or sequencing artifacts. Methodological guidance has therefore emphasized rigorous negative controls, careful sampling routes, contamination assessment, and standardized analytical procedures (18).

Detection of microbial DNA also does not establish microbial viability, persistent colonization, metabolic activity, or causal influence on implantation. Consequently, the IPT should not require the existence of a specific “healthy” endometrial microbiome as part of its core definition.

Microbial ecology is instead retained as an exploratory modifier that may interact with cytokine, growth-factor, epithelial, and immune signaling. This positioning is particularly important because emerging literature increasingly conceptualizes implantation as a coordinated network involving microbial, immune, endocrine, and growth-factor signals rather than isolated determinants. Any future microbial component of the IPT should therefore be tested using rigorous low-biomass microbiome methodology and integrated with host biological measurements rather than interpreted independently.

### 11.3 Seminal-fluid immunobiology: established activity, uncertain timing effect

Evidence that seminal fluid is biologically active within the female reproductive tract is substantially stronger than evidence concerning the effect of when exposure occurs. Seminal plasma contains cytokines, transforming growth factor-β, prostaglandins, and other signaling molecules capable of influencing epithelial responses, leukocyte recruitment, regulatory T-cell expansion, and reproductive immune adaptation (15, 16).

Human experimental evidence nevertheless remains limited. Catalini et al., (34) investigated vaginal seminal-plasma exposure in a randomized, double-blind, placebo-controlled design and identified enrichment of endometrial transcriptomic pathways related to immune activity, cellular proliferation and movement, implantation, embryo development, and angiogenesis. However, no individual genes remained significantly differentially expressed after multiple-testing correction, and the analyzed sample was small.

Thus, the evidence supports the proposition that seminal exposure can interact with female reproductive biology but does not establish that exposure during menstruation or before completion of the peri-menstrual immune transition is detrimental. The timing component introduced by the IPT should therefore remain an explicitly secondary and falsifiable interaction hypothesis.

This distinction also prevents conflation of seminal exposure with sexual behavior more broadly. If this interaction is tested empirically, studies should characterize exposure as precisely as possible and should account for contraception, semen exposure, frequency, cycle timing, infection risk, fertility intentions, and other relevant behavioral and biological factors.

### 11.4 Maternal immune activation and the limits of animal-to-human extrapolation

Maternal immune activation provides compelling experimental evidence that immune perturbation during pregnancy can influence placental and fetal development. However, the relevance of these models to the IPT is limited by major differences in exposure type, magnitude, timing, and anatomical context.

Many MIA models use polyinosinic:polycytidylic acid, lipopolysaccharide, infection, or cytokine administration to induce substantial systemic immune activation during established pregnancy (25–27, 45, 46). The IPT instead concerns physiological or subclinical variation in local endometrial immune–repair biology before implantation.

These models therefore demonstrate that maternal immune signaling can affect developmental biology under defined experimental conditions; they cannot be treated as validation of the proposed peri-menstrual pathway.

Human evidence presents additional challenges. Maternal inflammatory exposures are heterogeneous, and associations with neurodevelopment may be influenced by shared genetics, familial factors, maternal disease, medication exposure, infection severity, socioeconomic conditions, and other environmental variables (28). Stronger causal inference will require designs capable of addressing these sources of confounding rather than simply demonstrating statistical association.

### 11.5 Placental and epigenetic evidence: plausible downstream biology, not evidence of continuity

The placenta is a biologically plausible downstream mediator because implantation initiates trophoblast–decidual interactions that contribute to placental development. Contemporary single-cell studies reveal extensive ligand–receptor communication among trophoblast, stromal, vascular, epithelial, and immune-cell populations at the maternal–fetal interface (36).

Experimental perturbation can alter these networks. Lund et al. (37) demonstrated that lipopolysaccharide-induced inflammation altered cell composition and communication pathways at the maternal–fetal interface in an experimental model. Such findings establish sensitivity of this interface to inflammation but do not establish biological continuity from physiological peri-menstrual variation.

The same caution applies to epigenetic evidence. The peri-conceptional period involves extensive epigenetic reprogramming (24), but biological sensitivity does not imply that every upstream maternal exposure generates a persistent epigenetic signature. Furthermore, observed epigenetic differences may reflect cell composition, genetic background, later pregnancy exposures, or reverse causation.

Epigenetic mediation should therefore require a demonstrated exposure–outcome relationship, temporal precedence, exposure-associated molecular change, replication, and evidence that the molecular change contributes functionally to the downstream phenotype.

### 11.6 Autism and neurodevelopment: heterogeneity limits causal interpretation

Autism is not a biologically homogeneous outcome. Recent functional-connectivity research further supports the presence of biological heterogeneity within autism, including the identification of connectivity-based subtypes across human and cross-species analyses (47). Large genetic studies demonstrate contributions from common polygenic variation, rare inherited variants, and *de novo* variants, with substantial heterogeneity across individuals (31). Autism diagnosis is also a distal outcome occurring years after conception and is influenced by developmental surveillance, diagnostic practices, sex-related ascertainment, cognitive and language profiles, and healthcare access.

Environmental and pregnancy-related associations with autism are also heterogeneous and vulnerable to confounding. An evidence-based review of systematic reviews and meta-analyses identified varying levels of support across proposed environmental and pregnancy-related risk factors and emphasized the importance of prospective exposure measurement, developmental timing, and genetically informed approaches when evaluating environmental contributions to autism (48).

Older biomarker studies have additionally reported oxidative and DNA-damage abnormalities in some autistic individuals and their mothers (49). Such findings are nonspecific, do not establish directionality or causation, and provide no direct evidence for the peri-menstrual reproductive pathway proposed by the IPT.

These characteristics make autism a poor initial endpoint for testing the IPT. Even if a future association were observed, it would not establish that the proposed reproductive pathway is causal without demonstration of the intervening biological sequence and rigorous control for genetic and environmental confounding.

The revised framework therefore uses autism as an exploratory example within a broader neurodevelopmental domain rather than as the defining outcome of the hypothesis. Earlier dimensional developmental outcomes and neonatal biological measures would provide more proximal tests if the reproductive and placental components are first validated.

### 11.7 Bias and threats to causal inference

Future IPT research will face several major sources of bias.

Selection bias may arise because women planning pregnancy, undergoing assisted reproduction, or consenting to repeated reproductive sampling may differ from the general reproductive population.

Measurement bias may result from inaccurate menstrual timing, inconsistent definitions of bleeding cessation, assay variability, batch effects, sampling route, and imperfect surrogate measures of tissue-level biology.

Temporal misclassification is particularly important because endometrial immune and molecular characteristics change rapidly across the cycle.

Residual confounding may arise from maternal age, endocrine and metabolic status, infection, autoimmune disease, medication exposure, nutrition, smoking, environmental exposures, stress, socioeconomic conditions, embryo quality, paternal factors, and assisted-reproduction procedures.

Reverse causation should also be considered where embryonic or genetic characteristics influence maternal signaling rather than maternal biology acting unidirectionally on the embryo.

Multiple-testing and model-overfitting represent major risks in cytokine, transcriptomic, microbiome, metabolomic, and epigenomic analyses.

Finally, publication bias may preferentially amplify positive findings in emerging fields.

Prospective preregistration, repeated within-person sampling, blinded laboratory procedures, negative controls, independent replication, appropriate multiple-testing correction, and transparent reporting of null findings should therefore be considered central requirements rather than optional methodological refinements.

### 11.8 Overall critical assessment

Overall, the current literature provides a credible biological rationale for testing whether a reproducible peri-menstrual endometrial immune–repair phenotype exists and whether it prospectively predicts later receptivity and implantation. Preliminary human studies demonstrate the feasibility of obtaining biologically informative information from menstrual material, while established reproductive immunology provides a mechanistic basis for investigating later endometrial function.

However, the core IPT relationship remains unvalidated. Evidence becomes progressively weaker as the framework extends from receptivity and implantation toward seminal-exposure timing, placental persistence, epigenetic programming, and neurodevelopment. No direct evidence currently supports the complete proposed sequence.

The scientific strength of the IPT therefore lies not in claiming that these relationships are established, but in defining a hierarchy of experimentally separable questions and specifying the evidence that would support, modify, or refute each component.

## 12 Discussion

The Immunological Purity Theory proposes a testable framework for investigating whether measurable variation during the peri-menstrual endometrial immune transition has prospective relevance for subsequent receptivity and implantation. The principal contribution of the framework is therefore not the assertion of a new established causal pathway, but the identification of a specific and falsifiable question situated between menstrual immunobiology and later endometrial function.

The revised framework substantially narrows the central proposition. Menstruation and implantation are temporally separated, and the hypothesis does not assume persistence of menstrual inflammation until implantation. Instead, it proposes that measurable characteristics of the early immune–repair transition may reflect or predict the subsequent trajectory of endometrial regeneration, endocrine-dependent differentiation, and acquisition of the receptive phenotype. This distinction converts the proposed link from an assumed biological continuity into an empirical relationship that can be directly tested.

Preliminary human evidence supports the feasibility of investigating this relationship but does not validate it. Menstrual material can contain biologically informative molecular signals, and menstrual-blood-derived endometrial cells can retain functional characteristics relevant to endometrial differentiation and receptivity (11, 12). These observations justify prospective investigation of an early-cycle phenotype but do not establish that such a phenotype predicts implantation or has developmental consequences.

### 12.1 Conceptual contribution and operational refinement

A central refinement of the revised framework is the distinction between the conceptual name Immunological Purity Theory and the measurable biological construct peri-menstrual endometrial immune transition. This distinction addresses the risk that the term *purity* could be interpreted as absence of immune activity or inflammation. Normal reproductive physiology requires regulated inflammatory and immune processes, and the hypothesis does not propose a biologically immune-inactive endometrium.

Empirical evaluation should therefore focus on measurable variation in immune regulation, tissue repair, regeneration, vascular remodeling, and endocrine responsiveness rather than attempting to classify individuals as immunologically “pure” or “impure.” Whether these variables form a reproducible phenotype and whether that phenotype predicts later endometrial function are empirical questions.

This operational distinction also increases falsifiability. If no reproducible peri-menstrual phenotype can be demonstrated, the foundational premise is weakened. If such a phenotype exists but fails to predict later receptivity or implantation, the core reproductive hypothesis is not supported. The framework therefore does not depend on confirmation of every proposed downstream pathway.

### 12.2 Seminal exposure as a secondary interaction hypothesis

The revised framework also distinguishes the endogenous endometrial state from candidate modifiers of that state. Seminal exposure is biologically relevant because seminal fluid can induce inflammatory and regulatory responses within the female reproductive tract (15, 16). However, current evidence does not establish that exposure during menstruation or before completion of the proposed immune–repair transition is harmful.

The IPT therefore does not define intercourse timing as the primary causal exposure. Instead, it generates a secondary interaction hypothesis: the biological effects of seminal exposure may differ according to the endometrial immune–repair state at the time of exposure. The directional possibility that exposure before completion of an appropriately coordinated transition could be associated with a less favorable later receptivity phenotype is explicitly presented as a prediction requiring prospective testing.

This distinction is important clinically and conceptually. It preserves a testable question concerning exposure timing without converting an untested relationship into reproductive advice. It also permits the hypothesis to be refuted if seminal exposure timing shows no interaction with objectively measured endometrial state.

### 12.3 Reproductive outcomes precede developmental extensions

The revised framework places endometrial receptivity and implantation at the center of empirical validation. This prioritization reflects both biological proximity and evidential strength. If the proposed early-cycle phenotype does not predict later endometrial function, there would be little basis for attributing placental or neurodevelopmental significance to it.

Placental adaptation is therefore treated as a secondary mechanistic extension. The maternal–fetal interface involves extensive communication among trophoblast, stromal, vascular, epithelial, and immune-cell populations, and inflammatory perturbation can modify these networks under experimental conditions (36, 37). Nevertheless, such evidence demonstrates general biological sensitivity rather than continuity from peri-menstrual variation.

Epigenetic programming is positioned even further downstream. Although the peri-conceptional period is characterized by extensive epigenetic reprogramming (24), no evidence currently demonstrates that the proposed peri-menstrual phenotype produces persistent embryonic or placental epigenetic changes. Epigenetic analysis therefore becomes informative only after the proximal reproductive pathway is supported.

### 12.4 Neurodevelopment and autism

Neurodevelopment represents the most distal extension of the framework and should be interpreted accordingly. Maternal immune activation and developmental-programming research demonstrate that maternal biological conditions can influence developmental trajectories under particular circumstances, but these findings do not establish that subtle peri-menstrual endometrial variation produces comparable effects.

Autism is also a globally prevalent neurodevelopmental condition with substantial variation in reported prevalence across populations and study methodologies. A systematic review of global epidemiological studies reported a median prevalence of approximately 1% while emphasizing considerable geographic and methodological heterogeneity (50).

Autism spectrum disorder is a clinically defined, heterogeneous neurodevelopmental condition (51) and is especially unsuitable as an initial validation endpoint because of its substantial genetic contribution, etiological heterogeneity, long interval between conception and diagnosis, and susceptibility to multiple prenatal and postnatal influences (29–31).

Accordingly, the IPT does not propose that peri-menstrual immune variation or seminal exposure timing is an established cause of autism. Autism is retained as a distal exploratory outcome that could be investigated only if the preceding reproductive, placental, and developmental relationships receive sequential empirical support.

This distinction also means that the framework cannot currently explain differences in autism severity or presentation. Such questions would require dedicated studies examining developmental phenotypes, genetic liability, and relevant biological intermediates rather than extrapolation from reproductive immunology alone.

### 12.5 Scientific strengths and limitations

The principal strength of the IPT is that it integrates established but historically separated areas of reproductive biology into a sequence of testable questions. The framework identifies measurable proximal outcomes, distinguishes core from downstream hypotheses, incorporates competing explanations, and specifies conditions under which individual components would be weakened or refuted.

Its principal limitation is equally clear: direct evidence for the core IPT relationship is currently insufficient. Preliminary studies support the feasibility of measuring biologically informative signals during menstruation, but no adequately powered prospective study has demonstrated that a defined peri-menstrual immune–repair phenotype predicts later receptivity or implantation.

Additional limitations include the difficulty of measuring tissue-level biology non-invasively, substantial cycle-to-cycle variation, heterogeneity in receptivity assessment, confounding by endocrine and embryonic factors, uncertainty surrounding low-biomass endometrial microbiome measurements, and the increasing inferential distance between the core reproductive hypothesis and placental or neurodevelopmental outcomes.

These limitations are not resolved by conceptual integration alone. They require longitudinal human studies, repeated within-person sampling, standardized cycle staging, independent outcome measurement, replication, and appropriately controlled mechanistic experiments.

### 12.6 Clinical and ethical boundaries

The current framework does not justify recommendations regarding intercourse during menstruation, timing of conception, fertility treatment, menstrual practices, or prevention of autism or other neurodevelopmental conditions.

This limitation is particularly important because observational or theoretical reproductive research can be misinterpreted as assigning responsibility for developmental outcomes to maternal behavior. The IPT does not support such an interpretation. Reproductive and neurodevelopmental outcomes arise from complex interactions among maternal, paternal, embryonic, genetic, environmental, and stochastic factors.

Any future clinical application would require replicated evidence demonstrating a modifiable exposure, a causal relationship with a clinically meaningful outcome, and evidence that intervention improves outcomes without introducing harm. None of these criteria has yet been met for the IPT.

### 12.7 Historical context and hypothesis generation

Historical and religious traditions have long distinguished menstruation from a subsequent state of purification before the resumption of intercourse. In the Islamic tradition, Qur’an 2:222 specifically addresses menstruation and distinguishes the menstrual state from the subsequent state of purification before intercourse is resumed (52). The distinction contributed to the historical context from which the present research question was generated.

This historical observation is not presented as biological evidence for the Immunological Purity Theory, and the framework does not assume that a religious concept of purification corresponds to a defined immunological state. Rather, the scientific question generated from this historical distinction is whether an objectively measurable peri-menstrual endometrial immune transition exists and whether reproductive exposures—including seminal exposure—interact differently with the female reproductive environment according to the biological state at the time of exposure. The scientific validity of the hypothesis depends exclusively on empirical testing of these proposed relationships.

### 12.8 Overall interpretation

The appropriate interpretation of the Immunological Purity Theory at its current stage is therefore narrow but testable: does measurable variation in the peri-menstrual endometrial immune–repair transition prospectively predict later receptivity and implantation within the same cycle?

This question can be investigated independently of the more speculative downstream extensions. A positive answer would justify mechanistic studies of candidate modifiers, maternal–fetal interface biology, and developmental persistence. A negative answer would substantially weaken the central reproductive premise and would reduce the rationale for investigating more distal outcomes.

The scientific value of the framework therefore lies in its capacity to be progressively supported, refined, or rejected through empirical testing.

## 13 Conclusion

The Immunological Purity Theory proposes that measurable variation in the peri-menstrual endometrial immune transition may prospectively relate to subsequent endometrial receptivity and implantation. The revised framework does not assume persistence of menstrual inflammation, retained menstrual blood, or a direct causal pathway from menstruation to neurodevelopment. Instead, it defines an early-cycle immune–repair phenotype as the core empirical construct and asks whether this phenotype predicts the later functional state of the same endometrial tissue.

The hypothesis further identifies seminal exposure timing as a candidate effect modifier whose interaction with the peri-menstrual endometrial state requires direct testing. Current evidence establishes that seminal fluid is immunologically active within the female reproductive tract but does not demonstrate that peri-menstrual exposure is beneficial or harmful.

Placental adaptation, epigenetic programming, and neurodevelopment are retained as sequential downstream extensions rather than established consequences of the core reproductive hypothesis. No direct evidence currently links peri-menstrual endometrial immune variation or seminal exposure timing to autism spectrum disorder.

The immediate research priority is therefore to determine whether a reproducible peri-menstrual immune–repair phenotype exists and whether it prospectively predicts receptivity and implantation. Only after these proximal relationships are independently replicated should more distal placental and developmental outcomes be investigated.

The IPT should consequently be regarded as a falsifiable reproductive-immunology framework requiring empirical validation, not as a clinical explanation for autism or a basis for recommendations concerning reproductive timing.

## Data Availability

The original contributions presented in the study are included in the article/supplementary material. Further inquiries can be directed to the corresponding author.
